# Supplementary material for: WordCommentsAnalyzer: A windows software tool for qualitative research
Source: F1000Res. 2018 Sep 4;7:536. Originally published 2018 May 3. [Version 2] doi: 10.12688/f1000research.14819.2 (PMC6137411; doi:10.12688/f1000research.14819.2)
Supplement: Supplementary file 3 [file f1000research-7-17614-s0002.tgz › b2a79496-4ada-47c4-9682-a99ce867836d.zip!/Eight Iranian Health Journals/Yafteh _45.docx]

© Yafteh  . This work is licensed under CC BY 4.0 (https://creativecommons.org/licenses/by/4.0/).

Volume & Issue: دوره 9 - شماره 4

Title: بررسی میزان آگاهی مادران زائو نسبت به مزایای شیر مادر در شهر خرم آباد

http://yafte.lums.ac.ir/article-1-52-fa.html

مقدمه : شیر مادر به تنهایی تنها منبع تغذیه مناسب در 6 ماه اول زندگی است و در صورت تغذیه مناسب و کافی مادر در دوران شیر دهی ، انرژی و مواد مغذی مورد نیاز برای رشد و تکامل شیرخوار تأمین خواهد شد. اطلاعات و دانش ناکافی و یا باورهای غلط راجع به تغذیه شیرخوار ، شاغل بودن مادران و گرایش مادران شاغل به تغذیه شیرخوار با شیر خشک و یا شروع زودتر از موعد تغذیه تکمیلی، افزایش گرایش مادران به سزارین و فاصله افتادن بین نوزاد و مادر در ساعات طلائی اولیه شیر دهی و همچنین تبلیغات فریبنده در مورد شیر خشک همگی بخشی از علل کاهش گرایش به شیر مادر و افزایش استفاده از شیر خشک است . لذا هدف از این مطالعه بررسی میزان آگاهی مادران زائو در زمینه اهمیت تغذیه شیرخوار با شیر مادر بوده است. مواد و روش‌ها: در این مطالعه مقطعی، به منظور بررسی آگاهی مادران زائوی مراجعه کننده به بیمارستان حاج کریم عسلی خرم آباد در آذر ماه سال 1385 در زمینه اهمیت و مزایای تغذیه با شیر مادر کلیه زنان زائو در این فاصله زمانی مورد بررسی قرار گرفتند و پرسشنامه ای حاوی 17 سؤال بصورت تستی سه گزینه ای در اختیار مادران قرار می گرفت این سوالات در خصوص فوائد و اهمیت تغذیه شیرخوار با شیر مادر وهمچنین ترکیبات موجود در شیر مادر بود . پس از تکمیل پرسشنامه ها ، کد بندی متغیرها صورت گرفت و در نرم افزار کامپیوتری وارد گردید و میزان آگاهی مادران بر حسب هر یک از متغیرها با بکارگیری تستهای آماری مناسب سنجش گردید. . یافته‌ها: از مجموع 550 نفر مادر زائوی مراجعه تعداد 33 نفر (6%) میزان آگاهی ضعیف ، 216 نفر (39%) میزان آگاهی متوسط ، 301 نفر (55%) میزان آگاهی بالا داشتند . همچنین بین سن مادر و میزان آگاهی آنان ارتباط معنی داری وجود داشت به طوری که میانگین سن مادران با میزان آگاهی ضعیف حدود 26 سال ، مادران با آگاهی متوسط حدود 28 سال و مادران با آگاهی بالا حدود 31 سال بود . از طرفی مادران دارای تعداد فرزند بیشتر میزان آگاهی بیشتری داشتند . بین سطح سواد مادران و میزان آگاهی آنها ارتباط معنی داری وجود داشت به طوری که با افزایش میزان تحصیلات میزان آگاهی مادران در مورد اهمیت تغذیه با شیر مادر افزایش می یافت . در بین مادران شاغل میزان آگاهی بالاتر از مادران خانه دار بود و از نظر محل سکونت نیز مادران ساکن شهر میزان آگاهی بالاتری نسبت به مادران ساکن روستا داشتند . در این پژوهش همچنین مشخص گردید که بین سطح درآمد خانواده و میزان آگاهی مادران ارتباط معنی داری وجود ندارد . بحث و نتیجه‌گیری: با توجه به نتایج مطالعه مذکور، میزان آگاهی مادران تقریباً خوب می باشد ولی هنوز تعداد زیادی از مادران از آگاهیهای لازم در مورد تغذیه شیرخواران برخوردار نیستند . به ویژه مادران جوان و مادران ساکن روستا . لذا لزوم توجه بیشتر به این گروه از مادران ارائه برنامه های آموزشی مناسب در زمینه شیر مادر معطوف می شود .

Title: نیمرخ الکتروفورتیک آلبومین، آلفا 1، آلفا 2، بتا و گاماگلوبولین در سرم افراد وابسته و غیر وابسته به ترکیبات اپیوئیدی

http://yafte.lums.ac.ir/article-1-53-fa.html

مقدمه: مصرف ترکیبات اپیوئیدی در ایران از شیوع بالایی برخوردار است. آخرین رویکرد های پژوهشی در مورد سوء مصرف مواد نیز متوجه نقش مهم پروتئینها در شناخت ودرمانهای نوین این پدیده است. نظر به تاثیر استفاده طولانی مدت از ترکیبات اپیوئیدی بر عملکردکبد و ترکیب پروتئین های سرم انسان، این مطالعه با هدف بررسی طرح الکتروفورتیک پروتئینهای پلاسما در افراد وابسته به ترکیبات اپیوئیدی (تریاک وهروئین) طراحی واجراء گردید. مواد و روش‌ها: جمعیت مورد بررسی در این مطالعه مورد-شاهدی، شامل تعداد 42 نفر وابسته به تریاک، 35 نفر وابسته به هروئین و 35 داوطلب غیر وابسته به ترکیبات اپیوئیدی بودند که از نظر سن و جنس تطابق داشتند. مصرف ترکیبات اپیوئیدی در گروه های مورد، توسط تستهای تشخیص آزمایشگاهی ترکیبات اپیوئیدی در نمونه ادرار با استفاده از تست ایمنوکروماتوگرافی سریع و سپس تستهای تکمیلی کروماتوگرافی ستونی جامد- مایع و کروماتوگرافی نازک لایه تائید گردید. پس از خونگیری و تهیه سرم، الکتروفورز ناحیه ای انجام شد. داده ها با نرم افزار SPSS 11.5 آنالیز و به صورت Mean±SEM ارائه گردید. یافته‌ها: مقایسه میانگین مقدار آلبومین، - α 1 گلوبولین، 2 α – گلوبولین و بتاگلوبولین در بین سه گروه، تفاوت معنی داری را نشان نداد. اما مقدار گاماگلوبولین در گروه وابسته به تریاک (61/3 ± 38/17) و گروه وابسته به هروئین (4/4 ± 48/17) بطور معنی داری (01/0 p< ) بیش از گروه کنترل (8/1 ± 3/13 گرم در لیتر) بود. مقایسه مقادیر مذکور بین دو گروه وابسته به تریاک و وابسته به هروئین تفاوت معنی داری نشان نداد. بحث و نتیجه‌گیری: افزایش غلظت گاماگلوبولین ها میتواند در نتیجه تحریک سیستم ایمنی پس از اتصال ترکیبات اپیوئیدی و ناخالصیهای همراه با آنها به آلبومین یا به علت تحریک محور هیپوفیز-هیپوتالاموس باشد. هرچند که مصرف مداوم اپیوئیدها نیز تواماٌ بر سیستم ایمنی سلولی و هومورال تاثیر گذار است، معهذا افزایش معنی دار باند گاما میتواند ناشی از مصرف ترکیبات اپیوئیدی توام با رفتارهای پر خطر، ابتلا به بیماریهای عفونی و ناخالصی مواد افیونی باشد.

Title: بررسی اثر ضددردی و مواد متشکله عصاره برگ گیاه دارویی بن سرخ(Allium jesdanum) و نقش احتمالی سیستم اوپیوئیدی در اثرات ضددردی آن

http://yafte.lums.ac.ir/article-1-54-fa.html

مقدمه : درگزارش های اخیر خواص ضددردی گیاه دارویی بن سرخ با استفاده از آزمونهای فرمالین و Tail flick نشان داده شده است. بر همین اساس هدف این تحقیق بررسی و آنالیز مواد موجود در عصاره گیاه، آزمون حسی حرکتی و اثرات اوپیوئیدی قرار گرفت. مواد و روش‌ها: گیاه دارویی بن سرخ در ارتفاع 2200 متری منطقه سفید کوه لرستان در اردیبهشت ماه جمع آوری گردید و در دانشکده کشاورزی لرستان کد گزاری گردید. عصاره گیری با استفاده از دستگاه سوکسله انجام و سپس توسط روتاری حلال خارج گردید. در این مطالعه از120 عدد موش صحرایی نر نژاد Sprague-Dawely در محدوده وزنی 180-220 گرم و 5 0 عدد موش سوری در محدوده وزنی 20-2 5 گرم استفاده گردید. برای بررسی نقش سیستم اوپیوئیدی پیش تیمار با نالوکسان انجام شد و برای اندازه گیری درد از آزمون های Tail flick و Hot-Plate استفاده گردید. آزمون حسی حرکتی با استفاده از دستگاه روتارود انجام شد و آنالیز مواد متشکله با استفاده از دستگاه GC/ GC Mass انجام شد. یافته‌ها: نتایج نشان می دهد که تجویز داخل صفاقی عصاره هیدرواتانولی بن سرخ دارای اثرات ضد دردی می باشد که این اثرات با نالوکسان معکوس می شود. در دوزهای که عصاره دارای اثرات ضد دردی بود اثرات سمیت حرکتی از آن مشاهده نگردید. همچنین آنالیز مواد متشکله نشان داد که موادی نظیر مرفین سیلریت، اتیل سینامیت، ایزوکوینولین، نئومنتول و الکل های با زنجیر طولانی در عصاره وجود دارد. بحث و نتیجه‌گیری: به طور کلی می توان گفت بخشی از اثرات ضددردی عصاره مربوط به موادی است که بر سیستم اوپیوئیدی اثر می کند و این اثرات توسط نالوکسان از آنتاگونیست های مرفین معکوس می شود.

Title: بررسی اثر داروی کارنی‌تین (Carnitine) خوراکی در کاهش عوارض ناشی از همودیالیز مزمن، بخش دیالیز بیمارستان فاطمیه سمنان، تیر الی بهمن 1384

http://yafte.lums.ac.ir/article-1-55-fa.html

مقدمه: همودیالیز مزمن دارای هزینه بالایی هم برای بیمار و هم برای سیستم درمانی کشور است. در صورتی که بتوان عوارض حین دیالیز را به کمترین مقدار ممکن رساند منجر به کاهش هزینه‌های ناشی تکرار همودیالیز خواهد شد. ماده «ال کارنی ‌تین» ( L-Carnitine ) در عملکرد طبیعی عضلات اسکلتی و قلب ضروری است و شاید بتواند عوارض مختلف دیالیز را کاهش دهد. این مطالعه جهت بررسی نقش این ماده انجام شده است. مواد و روشها: بیماران بخش دیالیز بیمارستان فاطمیه در سال 1384 با استفاده از طراحی Cross-Over Clinical Trial و دوسوکور مورد بررسی قرار گرفتند. گروه اول دارونما و گروه دوم داروی ال‌کارنی‌تین خوراکی در حین دیالیز استفاده نمودند ( 500mg در روز). میانگین بروز عوارض مختلف دیالیز (افت فشارخون، کرامپ عضلانی و استفراغ) در هر جلسه دیالیز، در حین مصرف دارونما و ال‌کارنی‌تین خوراکی و مقایسه گردید. نتایج: از 33 مورد بیمار شرکت‌کننده در مطالعه، 26مورد دوره مطالعه را بپایان رساندند. بروز عوارض مختلف در حین مصرف دارو یا دارونما اختلاف معنی‌داری را نشان نداد. نتیجه گیری: بنظر می رسد مصرف این دارو در بیماران دیالیزی با دوز 500 mg در روز اثر مشخصی نداشته و باعث صرف هزینه زائد و تحمیل آن به بیمارمِی شود و لذا مصرف آن توصیه نمی گردد.

Title: بررسی فراوانی افسردگی و اضطراب در بیماران مبتلا به آلوپسی آره آتا مراجعه کننده به کلینیکهای تخصصی آموزشی و درمانی پوست و اعصاب و روان شهر خرم آباد

http://yafte.lums.ac.ir/article-1-56-fa.html

دریافت مقاله:14/4/86 ، پذیرش مقاله: 29/7/86 مقدمه : آلوپسی آره آتا یکی از علل شایع ریزش مو می باشد. این بیماری یک بیماری خود ایمنی است که با زمینه یک اختلال ژنتیکی و تحت تأثیر عوامل برانگیزاننده، عمدتاً استرس منجر به ریزش مو می شود. این بیماری می تواند باعث بروز مشکلات روحی و روانی بخصوص افسردگی و اضطراب گردد. مطالعه حاضر با هدف تعیین فراوانی افسردگی و اضطراب در بیماران مبتلا به آلوپسی آره آتا در شهر خرم آباد انجام گردید. مواد و روش‌ها: در این مطالعه توصیفی-مقطعی تعداد 80 بیمار مبتلا به آلوپسی آره آتا مراجعه کننده به کلینیکهای تخصصی آموزشی و درمانی پوست و اعصاب و روان به روش در دسترس، مورد بررسی قرار گرفتند. پس از ارجاع بیماران به روانپزشک با استفاده از تست روانشناسی 90- SCL موارد مشکوک شناسایی و با مصاحبه بالینی طبق معیارهای DSM-IV -IIIR تشخیص اضطراب و افسردگی قطعی می شد. داده ها با نرم افزار آماری 13- SPSS مورد تجزیه و تحلیل قرار گرفت. پس از تعیین فراوانی مطلق و نسبی، ارتباط متغیرها با آزمون مجذورکای انجام گردید . یافته‌ها: از مجموع 90 بیمار مورد بررسی 65% مرد، 8/53% در سنین کمتر از 25 سال و 5/67% مجرد بودند. 70% بیماران سابقه خانوادگی بیماری و 57% سابقه مصرف دارو نداشتند. در اکثریت موارد (8/63%) ریزش موی سر اتفاق افتاده بود. اکثریت بیماران مبتلا به آلوپسی آره آتا (1/80%) مبتلا به اضطراب و 1/75% مبتلا به افسردگی بودند که هم اضطراب و هم افسردگی در اکثریت بیماران شدید بوده (33% اختلال افسردگی شدید). آزمون آماری مجذورکای بین شیوع اضطراب و افسردگی با جنس ارتباط آماری معنی دار نشان د اد. در حالیکه با متغیرهای تحصیلات، تأهل، سابقه خانوادگی، سابقه مصرف دارو و محل شروع ضایعه ارتباط معنی دار به دست نیامد . بحث و نتیجه‌گیری: شیوع بالای اختلالات روانی در این بیماران شاخص می باشد. اضطراب، افسردگی، فوبیا، اختلالات خلقی و اختلالات سایکوتیک از جمله این اختلالات هستند که شیوع آنها در بیماران آلوپسی آره آتا از سایر مردم بیشتر بوده است. همچنین به مهارتهای سازگاری و ویژگیهای شخصیتی آنان مربوط باشد. نتایج نشان داد که شیوع اضطراب و افسردگی بخصوص از نوع شدید در زنان مبتلا به آلوپسی آره آتا بیش از مردان بوده است.این تفاوت را شاید بتوان ناشی از استرس زیبایی شناختی این بیماری به حساب آورد

Title: بررسی خصوصیات اپیدمیولوژیک و بالینی بیماران مبتلا به مولتیپل اسکلروزیس با شروع زودرس و مقایسه آن با موارد شروع شده در دوران بلوغ، شهر همدان سال 83-84

http://yafte.lums.ac.ir/article-1-57-fa.html

مقدمه : مولتیپل اسکلروزیس (MS) یکی از مهمترین بیماری‌های ناتوان کننده نورولوژیک در بالغین است. با این حال گزارشاتی دال بر بروز آن در سنین کمتر از 18سال وجود دارد که از آن تعبیر MS با شروع زودرس می‌شود. برای کسب اطلاعات دقیق‌تر در خصوص MS با شروع زودرس در غرب ایران، ویژگیهای اپیدمیولوژیک و بالینی بیماران مراجعه کننده به یک بیمارستان دانشگاهی در شهر همدان مورد بررسی قرار گرفته است. مواد و روش‌ها: در قالب یک مطالعه توصیفی تعداد 155 بیمار مبتلا به MS مراجعه کننده به بیمارستان سینا شهر همدان در طی سالهای1383 الی1384مورد بررسی قرار گرفتند.بیماران مطابق معیار Poser انتخاب شدند. علاوه بر آن ویژگی‌های بالینی و اپیدمیولوژیک بیماران مبتلا به شروع زودرس بیماری با موارد معمول آن مقایسه و مورد بررسی قرار گرفت یافته‌ها: در خصوص MS با شروع زودرس در این مطالعه،23 نفر دارای سن کمتر یا مساوی 18 سال بودند که از این بین 7 بیمار مرد و 16 بیمار زن بودند. میانگین سنی این گروه 63/16 ± 22/16 با محدوده 14 تا 18 سال بود. نوع بیماری MS در این گروه سنی به ترتیب17 بیمار پس‌رونده-عودکننده،5 بیمار پیشرونده ثانویه و یک بیمار پیشرونده اولیه بود. در هیچ یک از ویژگی‌های بالینی و اپیدمیولوژیک این بیماران تفاوتی آماری معنی ‌داری بین گروه بالغین و این گروه مشاهده نگردید. بحث و نتیجه‌گیری: مطالعه حاضر نشان دهنده تشابه ویژگی‌های اپیدمیولوژیک و بالینی بیماری MS در این مرکز دانشگاهی با سایر مراکز است. به جز آنکه در این مرکز موارد بیماری با شروع زودرس با شیوع بیشتری دیده می‌شود.

Title: بررسی مقایسه ای شیوع ژیاردیازیس در کودکان مدارس ابتدایی شهرستان دلفان

http://yafte.lums.ac.ir/article-1-58-fa.html

مقدمه : با توجه به شیوع بالای ژیاردیازیس در کودکان مدارس ابتدایی در اکثر مناطق کشور و لزوم تشخیص دقیق و سریع بیماری در آزمایشگاهها، مطالعه ای به منظور مقایسه روشهای معمول انگل شناسی در تشخیص ژیاردیازیس با روش نوین آنتی ژن یابی به کمک الایزاانجام گرفت. ضمن اینکه در کنار آن شیوع بیماری در منطقه دلفان از شهرستانهای استان لرستان نیز اندازه گیری شد. مواد و روش‌ها: این مطالعه یک مطالعه تحلیلی مقایسه ای است که بر روی 500 کودک دبستانی 12-6 ساله مدارس ابتدایی شهرستان دلفان انجام گرفت. پس از انتخاب نمونه ها به روش نمونه گیری تصادفی سیستماتیک به هر کدام از آنها فرم دعوتنامه اولیاء و مربیان داده شد تا ضمن شرح پروژه برای اولیاء، پرسشنامه های مربوطه نیز پر شده و روش نمونه گیری آموزش داده شود. از هر کودک سه نمونه مدفوع در سه نوبت متوالی گرفته شد. بر روی تمامی نمونه ها آزمایش گسترش مستقیم با سرم فیزیولوژی یا ید و تغلیظ فرمالین اتر انجام شده و نتایج حاصله ثبت گردید. سپس تکنیک الایزا بر روی نمونه ها انجام گرفته و نتایج بدست آمده با هم مقایسه گردید. یافته‌ها: از 500 کودک 6 تا 12 ساله دختر و پسر مدارس ابتدایی شهرستان دلفان در سال تحصیلی 83-1382 در مجموع 97 نفر در نتیجه انجام سه تکنیک گسترش مستقیم، تغلیظ فرمالین اتر و الایزا از نظر آلودگی به ژیاردیا لامبلیا مثبت شناخته شدند (4/19 %) که از این تعداد 68 نمونه (1/70 درصد) در تست گسترش مستقیم، 88 نمونه (72/90 درصد) در تست تغلیظ فرمالین اتر و 95 نمونه (93/97 درصد) با تکنیک الایزا مثبت شناخته شدند. بحث و نتیجه‌گیری: بر اساس نتایج حاصله از مطالعه حاضر تکنیک الایزا نسبت به روشهای روتین انگل شناسی در تشخیص ژیاردیا بسیار دقیق تر و سریع تر بوده، نیازی به افراد مجرب نداشته، در حضور کمترین مقادیر آنتی ژنهای انگل در مدفوع جواب داده و نیازی به ارگانیسم سالم نیز ندارد. با این تکنیک تعداد بیشتری نمونه را در مدت زمان کمتر می توان آزمایش نمود. شیوع بدست آمده از این مطالعه در مورد ژیاردیازیس در شهرستان دلفان نمایانگر لزوم آموزش بیشتر نکات بهداشتی در مدارس ابتدایی و تخصیص امکانات بهداشتی بیشتر در این منطقه می باشد.

Title: مقایسه روش رنگ آمیزی H&E با روش IHC در شمارش لنفوسیتهای

http://yafte.lums.ac.ir/article-1-59-fa.html

مقدمه : : از بیوپسی های دئودنوم بعنوان ابزاری برای غربالگری افراد مشکوک به بیماری سلیاک استفاده میشود. برای تشخیص این بیماری ، افزایش تعداد لنفوسیتهای اینترااپیتلیال (IELs) حتی در مواردیکه ساختمان پرزهای روده ای طبیعی است مهم بوده و می تواند دلیلی بر عدم تحمل به گلوتن باشد. هدف از این مطالعه مقایسه دو روش مختلف رنگ آمیزی بافتی H&E و IHC برای اندازه گیری لنفوسیتهای اینترااپیتلیال در بیوپسی های دئودنوم است مواد و روش‌ها: این مطالعه بصورت مقایسه ای و بر روی 74 نمونه بیوپسی دئودنوم از فروردین تا شهریور ماه سال 1383 در بیمارستان دکتر شریعتی تهران انجام پذیرفت. در این پژوهش همه لنفوسیتهای اینترااپی تلیال به روش یکسوکور مورد بررسی قرار گرفتند.. یافته‌ها: میانگین تعداد لنفوسیتهای اینترا اپیتلیال به ازای یکصد سلول اپیتلیال در مشاهده اول و دوم به ترتیب 77/15 و 72/16 برای نمونه های رنگ شده به روش H&E و 54/21 و 18/21 برای موارد رنگ شده به شیوه IHC محاسبه گردید. مقایسه میانگین لنفوسیتهای اینترا اپی تلیال در لامهای رنگ شده به روش H&E و LCA نشان داد که LCA در نشان دادن لنفوسیتها حدودا پنج سلول بر H&E برتری دارد. بحث و نتیجه گیری: برای آنالیز لنفوسیتهای اینترا اپیتلیال دو مرحله محاسبه لنفوسیتها با استفاده از رنگ آمیزی H&E ، رنگ آمیزی LCA و شمارش لنفوستها با این روش در مواردیکه پژوهشگر موارد مشکوک مواجه می شود ( موارد با لنفوسیتهای اینترا اپی تلیال نرمال اما نزدیک به نقطه cut – off point ) پیشنهاد می گردد. چنانچه در مواردی به هر دلیل رنگ آمیزی IHC انجام نشود میتوان از فرمول زیر استفاده کرد: IELs on H&E + 5 IELs on IHC .

Title: بررسی برخی مشکلات رفتاری ناشی از تنبیه بدنی توسط والدین درکودکان دبستانی

http://yafte.lums.ac.ir/article-1-60-fa.html

مقدمه: طرز رفتاربزرگسالان اثر عمیقی بر شخصیت کودکان و عادات کسب شده توسط آنها دارد. تنبیه بدنی به معنای استفاده از نیروی فیزیکی جهت ایجاد درد بدون صدمه، به منظور تربیت (تصحیح یا کنترل رفتار) کودک می باشد. تنبیه بدنی یک نوع کودک آزاری و شایع ترین نوع خشونت در خانواده است که موجب نقض حقوق کودک می شود. تنبیه بدنی دارای اثرات مخرب کوتاه و بلند مدت بر جسم و روح کودک می باشد. هدف از این پژوهش تعیین برخی مشکلات رفتاری در کودکانی که توسط والدین تنبیه بدنی می شوند و مقایسه آنها با کودکانی که توسط والدین تنبیه بدنی نمی شوند، می باشد. مواد و روش‌ها: پژوهش حاضر مطالعه ای توصیفی- تحلیلی است که روی240 کودک 12-7 ساله مشغول به تحصیل در مدارس ابتدائی خرم آباد در سال تحصیلی 84-1383 انجام شد. نمونه ها در دو گروه 120 نفری مورد (توسط والدین تنبیه بدنی می شدند) و شاهد (توسط والدین تنبیه بدنی نمی شدند) به روش تصادفی چند مرحله ای انتخاب شدند. ابزار جمع آوری اطلاعات پرسشنامه ای شامل 3 قسمت بود. بخش اول مشخصات فردی، بخش دوم سوالاتی در مورد تنبیه بدنی کودکان و بخش سوم شامل 20 سوال در ارتباط با مشکلات رفتاری پرخاشگری عملی، پرخاشگری کلامی، گوشه گیری و همکاری کودک در مدرسه بود. روش جمع آوری اطلاعات مصاحبه با کودک و معلم و مطالعه پرونده کودک درمدرسه بود. اطلاعات جمع آوری شده با استفاده از نرم افزار SPSS ver. 11 مورد بررسی و تجزیه تحلیل قرارگرفت. یافته‌ها: نتایج نشان داد نحوه تنبیه در 6/92% سیلی زدن به صورت کودک بود. بین دو گروه از نظر تحصیلات مادر (001/0= p ) و شغل مادر (004/0= p )، رتبه تولد کودک (05/0 P< )، پر خاشگری عملی (001/0 P< ) ، پرخاشگری کلامی (001/0 P< ) ، گوشه گیری (05/0 P< ) و همکاری((001/0 P< ) تفاوت آماری معنی داری به دست آمد . بحث و نتیجه‌گیری: نتایج نشان داد مادران خانه دار و دارای سطح تحصیلات پایین تر بیشتر از تنبیه بدنی استفاده می کنند و مشکلات رفتاری مثل پرخاشگری عملی و کلامی، گوشه گیری و عدم همکاری در کودکانی که توسط والدین تنبیه می شدند نسبت به آنها که تنبیه نمی شدند به طور معنی داری بیشتر بود. آموزش اثرات بد تنبیه بدنی بر جسم و روح کودک به والدین و نیز آموزش روشهای تربیتی مثبت به آنها می تواند در کاهش استفاده از تنبیه بدنی به عنوان یک روش تربیتی موثر باشد. پرستاران کودک و بهداشت جامعه در صورت داشتن مهارت و اطلاعات لازم می توانند در این زمینه نقش بسیار موثری ایفا کنند.

Title: اثر تیتانیم بر جذب اسید اولئیک در آنتروسیتهای EGS رت

http://yafte.lums.ac.ir/article-1-61-fa.html

مقدمه: عناصر مختلف قادر به تداخل با جذب اسیدهای چرب هستند. املاح تیتانیم امروزه به عنوان رنگدانه رنگ سفید در ساخت بسیاری از محصولات صنعتی از جمله پلاستیک، جوهر کاغذ، سرامیک، داروها، مواد آرایشی، غذاها و رنگسازی بکارمیرود. اخیرا عوارض جانبی این عنصر بر متابولیسم سلولی مشخص و گزارشات متعددی از اثرات سمی این ماده منتشر گردیده است . مواد و روش‌ها: ابتدا از روده کوچک رات نژاد ویستار به مقدار کافی ( (EGS Everted Gut Sac تهیه گردید. سپس EGS تهیه شده در محلول انکوباسیون حاوی اسیداولئیک در شرایط مختلف از نظر ph دما و غلظتهای مختلف تیتانیم قرار گرفته و اسیداولئیک انتقال یافته از غشاء EGS به داخل به روش اسپکتروفتومتری اندازه گیری گردید. اطلاعات بدست آمده با نرم افزار SPSS وآزمون ttest ارزیابی شد یافته‌ها: نتایج بدست آمده نشان میدهد که عواملی چون غلظت اسید اولئیک، زمان انکوباسیون و ph بر روی انتقال اسید اولئیک از غشاء مؤثر بوده است. بر اساس نتایج بدست آمده غلظتهای 5/0، 1، 5/1، 5، 10 میکرومولار تیتانیم در حضور کلرید سدیم بترتیب باعث کاهش 14.6%، 31.6%، 38.6% و 54.5% جذب اسید اولئیک می شود. استفاده از غلظتهای 0.5، 1، 1.5و 10 میکرومولار تیتانیوم در عدم حضور کلرید سدیم بترتیب باعث کاهش 5/3%، 28%، 29% و 39% جذب اسید اولئیک می شود. بحث و نتیجه‌گیری: نتایج بدست آمده نشان میدهد که عواملی چون غلظت اسید اولئیک، زمان انکوباسیون و ph بر روی انتقال اسید اولئیک از غشاء مؤثر بوده است. بر اساس نتایج بدست آمده تیتانیم باعث کاهش جذب اسید اولئیک از غشاء انتروسیتها شده است. این نتیجه در مورد افرادی که در معرض غلظتهای بالای این عنصر قرار می گیرند مهم بوده وباید همواره مد نظر قرار گیرد.

Title: گزارش دو مورد اداره بیهوشی دربیماری اپیدرمولیزبولوزا

http://yafte.lums.ac.ir/article-1-62-fa.html

مقدمه : بیهوشی در بیمار اپیدرمولیز بولوزا چالش بزرگی حتی برای با تجربه ترین متخصص بیهوشی می باشد. چراکه در این بیماری فشار غیر مستقیم سبب ایجاد تاول و زخم در غشائ مخاطی و پوست بدن می گردد . معرفی مورد: در این مقاله به معرفی نحوه اداره بیهوشی در دو مورد بیمار مبتلا به دیستروفیک اپیدرمولیزبولوزامی پردازیم که جهت انجام عمل جراحی پیوند پرده آمنیون بر روی چسبندگی های انگشتان به اتاق عمل آورده شدند. بحث و نتیجه‌گیری: در اداره بیهوشی این بیماران در زمانی که اقدام جراحی نیاز به شل کننده عضلانی و تهویه مکانیکی ندارد، برای اجتناب از دستکاری راه هوایی کتامین توصیه می شود.
